# Elevated Levels of Peripheral Kynurenine Decrease Bone Strength in Rats with Chronic Kidney Disease

**DOI:** 10.3389/fphys.2017.00836

**Published:** 2017-10-31

**Authors:** Bartlomiej Kalaska, Krystyna Pawlak, Tomasz Domaniewski, Ewa Oksztulska-Kolanek, Beata Znorko, Alicja Roszczenko, Joanna Rogalska, Malgorzata M. Brzoska, Pawel Lipowicz, Michal Doroszko, Anna Pryczynicz, Dariusz Pawlak

**Affiliations:** ^1^Department of Pharmacodynamics, Medical University of Bialystok, Bialystok, Poland; ^2^Department of Monitored Pharmacotherapy, Medical University of Bialystok, Bialystok, Poland; ^3^Department of Toxicology, Medical University of Bialystok, Bialystok, Poland; ^4^Faculty of Mechanical Engineering, Institute of Biocybernetics and Biomedical Engineering, Bialystok University of Technology, Bialystok, Poland; ^5^Department of Mechanics and Applied Computer Science, Faculty of Mechanical Engineering, Bialystok University of Technology, Bialystok, Poland; ^6^Department of General Pathomorphology, Medical University of Bialystok, Bialystok, Poland

**Keywords:** chronic kidney disease, bone disorders, tryptophan metabolites, kynurenine pathway, aryl hydrocarbon receptor

## Abstract

The diagnosis and treatment of bone disorders in patients with chronic kidney disease (CKD) represent a clinical challenge. CKD leads to mineral and bone complications starting early in the course of renal failure. Recently, we have observed the positive relationship between intensified central kynurenine turnover and bone strength in rats with subtotal 5/6 nephrectomy (5/6 Nx)-induced CKD. The aim of the present study was to determine the association between peripheral kynurenine pathway metabolites and bone strength in rats with 5/6 Nx-induced CKD. The animals were sacrificed 1 and 3 months after 5/6 Nx or sham operation. Nephrectomized rats presented higher concentrations of serum creatinine, urea nitrogen, and parathyroid hormone both 1 and 3 months after nephrectomy. These animals revealed higher concentrations of kynurenine and 3-hydroxykynurenine in the serum and higher gene expression of aryl hydrocarbon receptor (AhR) as a physiological receptor for kynurenine and AhR-dependent cytochrome in the bone tissue. Furthermore, nephrectomy significantly increased the number of osteoclasts in the bone without affecting their resorptive activity measured in serum. These changes were particularly evident in rats 1 month after 5/6 Nx. The main bone biomechanical parameters of the tibia were unchanged between nephrectomized and sham-operated rats but were significantly increased in older compared to younger animals. A similar trend was observed for geometrical parameters measured with calipers, bone mineral density based on Archimedes' method and image of bone microarchitecture obtained from micro-computed tomography analyses of tibial cortical bone. In nephrectomized animals, peripheral kynurenine levels correlated negatively with the main parameters of bone biomechanics, bone geometry, and bone mineral density values. In conclusion, our data suggest that CKD-induced elevated levels of peripheral kynurenine cause pathological changes in bone structure *via* AhR pathway. This finding opens new opportunities for the treatment/prevention of osteoporosis in CKD.

## Introduction

Chronic kidney disease (CKD) is a worldwide serious public health problem affecting ~7% of adults over age 30; the prevalence of at least moderate CKD in the population aged 65 and older is estimated to be about 30% (Levey et al., 2007; Zhang and Rothenbacher, 2008). Patients with CKD have complex amino acids alterations (Kopple, 1978; Zhao, 2013). Several exogenous and endogenous amino acid metabolites are accumulated in blood in the course of CKD. The significant disturbances occur in the peripheral tryptophan (TRP) degradation *via* kynurenine pathway, which resulted in the significant decrease of plasma TRP levels and augmentation of the kynurenine pathway metabolites (Pawlak et al., 2001a). Disturbances in the kynurenine pathway may contribute to uremic symptoms such as anemia (Pawlak et al., 2003), neurological disorders, and increased vulnerability to infections (Heyes et al., 1992; Li et al., 2007; Pawlak et al., 2009).

Changes in bone metabolism are common in CKD patients and have been classified as a new systemic disorder known as CKD-mineral and bone disorder (CKD-MBD) (Moe et al., 2009). CKD-MBD is characterized by calcium and phosphorus abnormalities, secondary hyperparathyroidism, vitamin D deficiency, vascular calcification, and bone abnormalities. It causes morbidity, mortality, increased the risk of fractures, and decreased the quality of life (Moe et al., 2006). Despite increasingly sophisticated diagnostic techniques for CKD-MBD, the pathophysiology of this disorder raises many doubts due to its complexity (Hruska et al., 2017). Our recent study indicates that the elevated concentrations of peripheral serotonin, a monoamine synthesized from tryptophan, correlates inversely with bone stiffness in growing rats with experimental CKD (Pawlak et al., 2016). In bone biology, peripherally synthesized serotonin inhibits bone formation. On the other hand, serotonin, when produced centrally, enhances bone formation, and limits bone resorption (Ducy and Karsenty, 2010). Both central and peripheral serotonin is produced from the TRP. Besides incorporation into protein, ~1% of ingested TRP is converted into serotonin, whereas the majority of TRP is metabolized along the kynurenine pathway. The kynurenine pathway leads to the production of many biologically active molecules such as kynurenine (KYN), 3-hydroxykynurenine (3-HKYN), xanthurenic acid, anthranilic acid, 3-hydroxyanthranilic acid, picolinic acid, and quinolinic acid (Schwarcz, 2004; Kalaska et al., 2016).

The knowledge of the role of the degradation products of TRP *via* the kynurenine pathway in bone metabolism is limited. Apalset et al. (2014) reported that higher bone mineral density is associated with high serum concentrations of two kynurenines: xanthurenic acid and 3-hydroxyanthranilic acid. Patients with osteoporosis had lower concentrations of TRP and 3-hydroxyanthranilic acid, whereas higher levels of anthranilic acid (Forrest et al., 2006). Recently, Vidal et al. (2015) observed a substantial increase in TRP degradation *via* kynurenine pathway during osteoblastogenesis. Authors found the potent inhibition of osteoblastogenesis after blocking of indoleamine 2,3-dioxygenase type 1, the enzyme catalyzing the degradation of TRP in the kynurenine pathway. Recently published data suggest that dietary and intraperitoneally administered kynurenine accelerates age-related bone loss by impairing osteoblastic differentiation and increasing osteoclastic resorption (Refaey et al., 2017).

Recently, we observed the positive effect of central kynurenine on bone strength in rats with CKD (Kalaska et al., 2017). In the current study, we investigated the role of peripheral TRP catabolism, aryl hydrocarbon receptor (AhR) as a physiological receptor for KYN and AhR-dependent cytochrome P450 1A1 (CYP1A1) in the development of osteoporosis in CKD. We performed the experiments on Wistar rats with nephrectomy-induced CKD. We used a wide range of non-invasive and invasive testing methods to provide the comprehensive understanding of bone quality in rats with CKD. Our methods included techniques for characterization of bone histology, geometry, microarchitecture, and mechanical properties, as well as bone remodeling markers. Histologic analysis provides unique information on bone remodeling and pathophysiology of the bone disease. Micro-computed tomography (micro-CT) is used for evaluation of bone microarchitecture and morphology. Furthermore, measurement of bone stiffness and strength using three-point bending allows direct assessment of a range of mechanical properties allowing characterization of multiple material and structural properties (Moe et al., 2009; Donnelly, 2011). In our study, we focused on the relationship between peripheral TRP and its metabolite *via* kynurenine pathway and mechanical and geometrical indicators of the bone. The study was based on the hypothesis that disturbances in peripheral kynurenine pathway may affect bone metabolism and strength by AhR signaling in the course of CKD.

## Materials and methods

### Animals and housing

Forty male Wistar rats were purchased from the Center of Experimental Medicine in Medical University of Bialystok (Poland). Animals were housed in temperature and humidity controlled room according to Good Laboratory Practice rules. A health surveillance program monitored the animals' health status according to Federation of European Laboratory Animal Science Associations guidelines. All the procedures involving rats were approved by Local Ethical Committee at the Medical University of Bialystok (Permit Number 17/2012). The study was conducted by ARRIVE guidelines (Kilkenny et al., 2010) and directive 2010/63/EU of the European Parliament and of the Council on the protection of animals used for scientific purposes. Exsanguination euthanized all animals at the end of the experiment.

### Design of experiment

Wistar rats weighing 114 ± 15 g and aged 4 weeks were randomly divided into four groups: sham-operated rats, biological material collected 1 month after surgery (Sham-1, *n* = 8); subtotal nephrectomized rats, biological material collected 1 month after surgery (5/6 Nx-1, *n* = 12); sham-operated rats, biological material collected 3 months after surgery (Sham-3, *n* = 8); and subtotal nephrectomized rats, biological material collected 3 months after surgery (5/6 Nx-3, *n* = 12). The subtotal nephrectomized rats underwent surgical resection of 5/6 kidney in two-steps according to the procedure described by Sviglerova et al. (2010). Sham-operated rats experienced renal evacuation and decapsulation and then the intact organ return into the abdominal cavity. One or three months after second surgery, rats were weighed, anesthetized until unconscious and blood samples were taken from cardiac puncture. After centrifugation, serum was stored and frozen −80°C until assays. Kidneys were removed, photographed, weighted using electronic scales Kern ALT 100-5-A (Germany), and illustrated with Masson trichrome staining. Left tibias were dissected, cleaned of adhering tissue, weighted using Kern ALT 100-5-A (Germany), measured with calipers (Artpol, Poland), wrapped in saline-saturated gauze, and frozen −20°C until biomechanical and geometrical analysis. Right tibias were dissected, cleaned of adhering tissue, and fixed in 4% neutral formalin for histological examination (*n* = 8 from each group) or frozen −80°C until micro-CT analysis (*n* = 4 from each group). The gene expression of AhR and CYP1A1 in the proximal part of the right tibia were also determined.

### Serum biochemistry

The serum urea, creatinine, sodium, and potassium concentrations were measured with the commercially available kit (CORMAY, Poland) using biochemical analyzer Minidray BS-120 (USA). The inorganic phosphorus and calcium were measured by Phosphorus and Calcium arsenazo kits (BioMaxima, Poland). Intact parathyroid hormone (PTH) was determined by ELISA (Immunotopic, CA, USA). Alkaline phosphatase (ALP) and osteoclast-derived tartrate-resistant acid phosphatase form 5b (TRACP 5b) activities were measured using commercially available colorimetric kits purchased from BioMaxima (Poland) and Immunodiagnostic Systems (Germany), respectively.

### Kidney histology

The harvested kidneys were fixed in 4% formalin. Renal tissues were cut into 4 μm thick sections and stained with Masson trichrome for histological analysis. A blinded observer performed a semiquantitative evaluation based on percentages. Assessment of glomerulosclerosis and the tubular injury was performed using the semiquantitative scale described by Cao et al. (2000).

### Bone biomechanics

**Figure 1:**
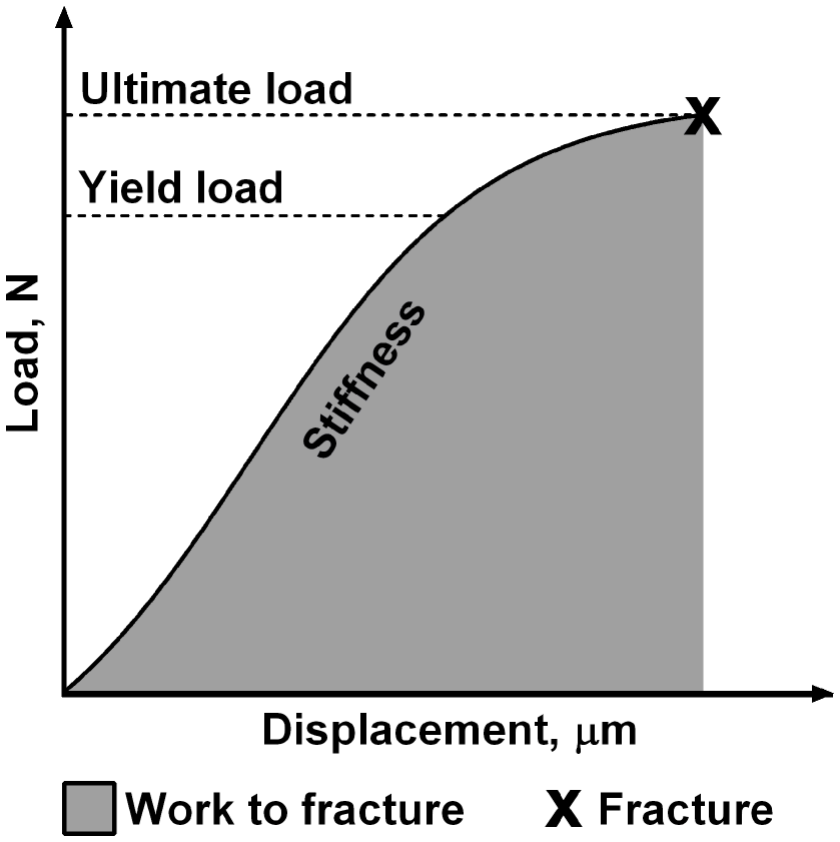
The schematic presentation of mechanical properties of the individual bone obtained in the three-point bending test.

Before biomechanical testing, left tibias were thoroughly thawed to room temperature. The biomechanical properties of left tibias were determined using the three-point-bending test as described previously (Brzoska et al., 2005; Kalaska et al., 2017). The testing was performed with a testing machine Zwick Roell Z.2.5 (Germany) using testXpert II software. A load was applied bone midway between two supports separated by a constant distance and the bending occurred at the medial-lateral axis. Cortical load and displacement values were recorded during the test, and the load-displacement plot was generated for analysis. Four parameters that describe the bone structural properties were obtained from this plot (Figure 1). Each parameter reflects different biomechanical properties of the individual bone. Stiffness is defined as the resistance of whole bone to the applied load. Yield load is a measure of the force that does not cause the permanent bone damage. Ultimate load is the maximum value of load attained during the bending test which is necessary to cause the fracture of the bone. Moreover, work to fracture represents the work that must be performed to fracture the bone (Turner, 2006; Oksztulska-Kolanek et al., 2016).

### Bone cross-sectional geometry

After biomechanical testing, the same fragments of broken bones were measured with calipers to obtain anterior-posterior periosteal diameter, medial-lateral periosteal diameter, anterior-posterior endosteal diameter, medial-lateral endosteal diameter, and wall thickness. Cortical index, cross-sectional area, mean relative wall thickness and cross-sectional moment of inertia were calculated using formulas described previously (Brzoska et al., 2005; Gajos-Michniewicz et al., 2012).

### Bone mineral density

Volumetric bone mineral density was measured based on Archimedes' principle (Keenan et al., 1997) using the automatic balance (RADWAG AS 60/220R2, Polska).

### Bone histology

After the decalcification according to Richman et al. (1947), the proximal tibias were cut in the antero-posterior planes and fixed for 1 h in 4% neutral buffered formalin. Paraffin-embedded blocks were cut into 4 μm thick sections and stained with hematoxylin and eosin. The osteoclasts number was rated per millimeter of the bone perimeter in 10 of randomly selected places in the secondary spongiosa of the proximal tibia of each animal, starting between 1 and 2 mm distal to the growth plate according to Miller et al. (1998). Osteoclasts were defined as large-shaped cells attached to the bone interface.

### Quantitative analysis using micro-CT

Dissected tibias were placed in styrofoam tube covered with stretch foil during scanning to prevent drying. Tibias were scanned using the 1172 SkyScan microCT desktop scanner (SkyScan, Kontich, Belgium) and subsequently reconstructed and analyzed with the packaged NRecon and CTAn software. The X-ray source was operated at 80 kV/124 μA with a 0.5 mm Al filter. Images were acquired at a 5.44 μm resolution with a 0.4° rotational step. Scans were reconstructed with 20% beam hardening and ring correction factor of 6. From the reconstructed datasets, the cortical volume of interest (VOI) was defined for each tibia. The growth plate cross-section served as an anatomical reference from which the long axis of VOI was defined. The cortical VOI extended from 1,500 to 1,700 slices (8.16–8.98 mm) relative to the growth plate. Cortical VOIs were thresholded at 90, on an 8-bit grayscale, and 2D and 3D histomorphometric parameters were obtained (Bouxsein et al., 2010). Cortical bone density (BMD Ct) was calculated with the use of 4 mm diameter bone density phantoms (Bruker-MicroCT BMD calibration phantoms) used to mimic rat tibias. Cortical bone morphometric parameters: cortical bone volume fraction (BV/TV), cortical thickness (Ct.Th), total cross-sectional area (Tt.Ar), cortical bone area (Ct.Ar), cortical area fraction (Ct.Ar/Tt.Ar) and marrow area (Ma.Ar) were analyzed. Additionally, data about closed porosity [Po(cl)] and volume of closed pores [Po.V(cl)] were gathered.

### High-performance liquid chromatography (HPLC)

Serum concentrations of TRP and its metabolites *via* kynurenine pathway were determined by HPLC (Agilent Technologies 1260 series LC system). Deproteinized serum samples were prepared by adding 2M perchloric acid. Then, the acidified samples were vortexed, kept at 4°C for 10 min, and centrifuged at 14,000 × g for 30 min at 4°C. The supernatant was injected into HPLC system for analysis. TRP and KYN were determined according to Holmes (1988). 3-HKYN was determined according to Heyes and Quearry (1988). Serum concentrations of TRP, KYN, and 3-HKYN were expressed in μM, μM, and nM, respectively.

### Quantitative real-time polymerase chain reaction (qRT-PCR) assay

Total RNA was isolated with the Thermo Scientific GeneJET RNA Purification Kit (Thermo Scientific, Lithuania) according to manufacturer's instructions. Quantification and quality control of RNA was checked on an Agilent 2100 Bioanalyzer (Agilent Technologies). An aliquot of 1 μg of total RNA was reverse transcribed with the RevertAid™ First Stand cDNA Synthesis Kit (Fermentas, Canada). QRT-PCR was performed using the Stratagene Mx3005P QPCR System (Agilent Technologies, USA) with the SG qPCR Master Mix (2x) (EURx, Gdansk, Poland). Reactions were run in duplicate and contained 2 μL of cDNA template along with 0.3 μM primers in a final reaction volume of 25 μL. Cycling parameters were 95°C for 10 min to activate DNA polymerase, then 40 cycles of 95°C for 15 s and 60°C for 30 s, with a final recording step of 72°C for 25 s to prevent any primer-dimer formation. Reactions were checked by including no-RT-controls, by the omission of templates and by both melting curves to ensure only the single product was amplified. Primers were designed using Primer-BLAST software (http://www.ncbi.nlm.nih.gov/tools/primer-blast). The primer sequences were (5′-3′ forward, reverse): AhR, ACAGTTTTCCGGCTTCTTGC, GTTCGCGTCCTTCTTCATCC; CYP1A1, AGTTCAGTCCTTCCTCACAGC, TGAAGGCTGGGAATCCATACA; glyceraldehyde 3-phosphate dehydrogenase (GAPDH), AAGATGGTGAAGGTCGGTGT, AGGTCAATGAAGGGGTCGTT. Relative quantification of gene expression was determined by comparison values of Ct using the ΔΔCt method. All results were normalized to GAPDH.

### Statistical analysis

Shapiro-Wilk's test was used for data distribution analysis. The normally distributed data were analyzed using a two-way analysis of variance (ANOVA) and shown as mean ± *SD*. The two independent factors were: age (1 or 3 months) and group (Sham or 5/6 Nx). If the results of ANOVA showed significant differences (*p* < 0.05), *post-hoc* Bonferroni test was used to verify the level of significance between individual groups. The non-Gaussian data were presented as median (full range) and analyzed using the non-parametric Mann-Whitney test. The correlations between variables were calculated by Spearman's rank or Pearson's correlation analyses. A two-tailed *p* < 0.05 was considered statistically significant. The data were analyzed using Statistica 12 computer software (StatSoft, USA). Graphic design presentation of results was performed using GraphPad Prism 6 (USA) or R statistical software (version 3.3.2).

## Results

### General characteristics of rats

Table 1 shows the body weight, biochemical parameters, and bone turnover biomarkers in sham-operated and nephrectomized rats. Final body weight and body weight gain were significantly lower in the 5/6 Nx-3 group than in the Sham-3 group. The 5/6 Nx groups presented higher serum concentrations of creatinine and urea nitrogen compared to healthy controls. Nephrectomized animals also developed hyperparathyroidism. The similar serum concentrations of phosphorus, calcium, sodium, and potassium were observed in all studied groups. There were also no differences in the serum ALP and TRACP 5b activities between 5/6 Nx and controls.

**Table 1:** Body weight and biochemical parameters in sham-operated (Sham) and nephrectomized (5/6 Nx) rats after one (Sham-1 and 5/6 Nx-1) and 3 months (Sham-3 and 5/6 Nx-3) of disease progression.

	**Sham-1**	**5/6 Nx-1**	**Sham-3**	**5/6 Nx-3**
Final body weight, g	275.5 ± 11.1	254.8 ± 13.5	391.1 ± 27.4^∧∧∧^	338.4 ± 13.6^***^^,^^###^
Weight gain, g	145.5 ± 14.3	141.7 ± 12.1	277.0 ± 44.1^∧∧∧^	230.2 ± 15.7^***^^,^^###^
Creatinine, mg/dL	0.31 ± 0.03	0.51 ± 0.11^***^	0.39 ± 0.04	0.60 ± 0.06^***^^,^^##^
Blood urea nitrogen, mg/dL	50.1 ± 5.2	80.5 ± 12.5^***^	42.1 ± 4.0	71.4 ± 10.1^***^
Phosphorus, mg/dL	6.26 ± 2.21	6.31 ± 2.33	6.38 ± 1.35	6.22 ± 2.36
Calcium, mg/dL	5.56 ± 1.86	5.21 ± 1.79	6.12 ± 1.77	4.16 ± 0.80
Sodium, mmol/L	147.9 ± 1.4	147.3 ± 2.1	146.4 ± 2.3	146.7 ± 2.9
Potassium, mmol/L	5.69 ± 0.24	5.96 ± 0.41	5.79 ± 0.46	6.43 ± 0.92
PTH, pg/mL	220.7 ± 75.2	387.1 ± 85.3^*^	368.1 ± 55.5	579.7 ± 183.9^**^^,^^##^
ALP serum, U/L	60.7 ± 22.8	68.3 ± 25.3	31.9 ± 9.1^∧^	31.1 ± 7.3^###^
TRACP 5b serum, U/L	172.7 ± 24.1	227.7 ± 79.8	144.8 ± 32.6	177.1 ± 43.6

* *p < 0.05*,

** *p < 0.01*,

*** p < 0.001 vs. appropriate sham group;

∧ *p < 0.05*,

∧∧∧ p < 0.001 vs. Sham-1;

## *p < 0.01*,

### *p < 0.001 vs. 5/6 Nx-1, ANOVA with post-hoc Bonferroni correction. Data are mean ± SD. PTH, parathyroid hormone; ALP, alkaline phosphatase; TRACP 5b, tartrate-resistant acid phosphatase form 5b*.

### Kidney macroscopy and histology

**Figure 2:**
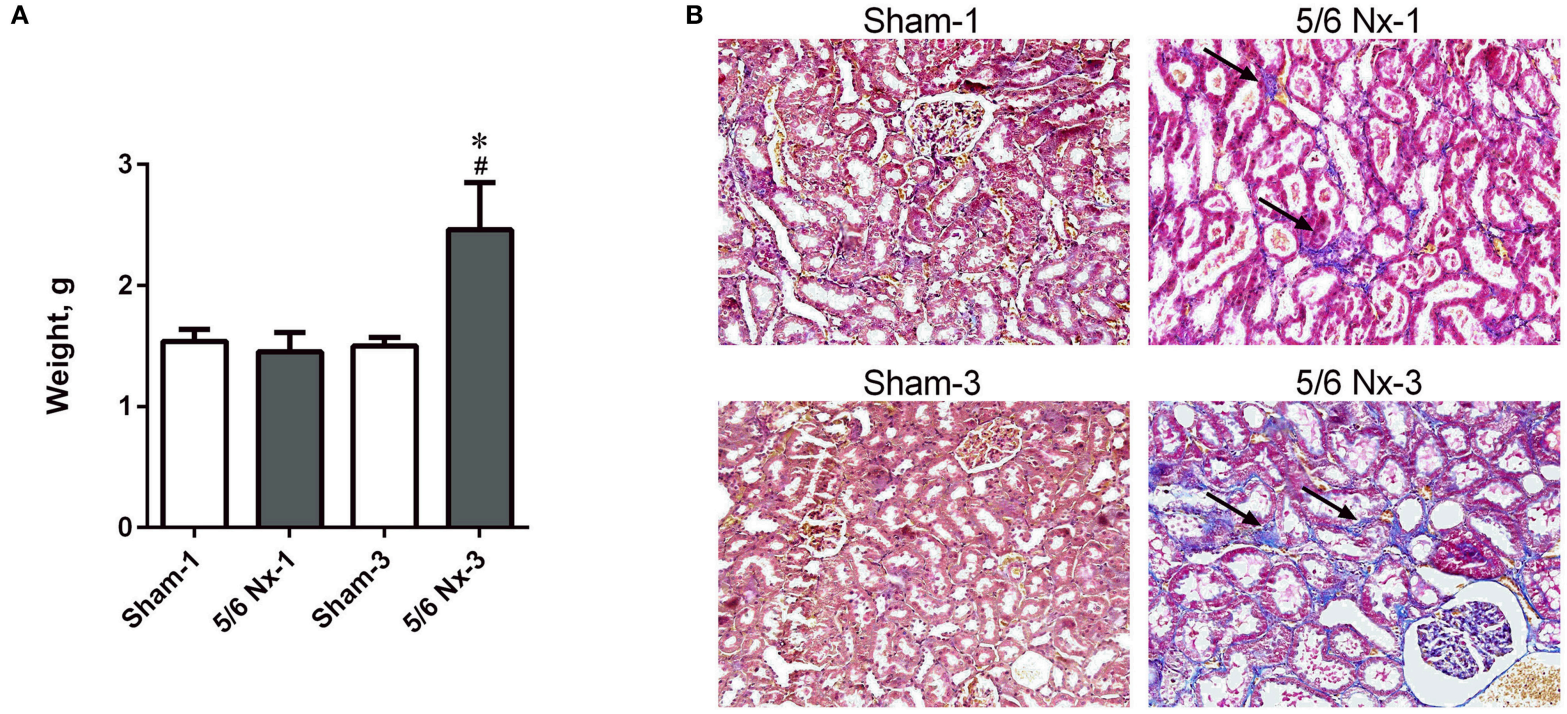
The weight of the remnant kidney **(A)** and histological changes **(B)** in the example kidney taken from sham-operated (Sham) and nephrectomized (5/6 Nx) rats after one (Sham-1 and 5/6 Nx-1) and 3 months (Sham-3 and 5/6 Nx-3) of disease progression. ^*^p < 0.05 vs. appropriate sham group; ^#^*p* < 0.05 vs. 5/6 Nx-1, ANOVA with *post-hoc* Bonferroni correction. Data are mean ± *SD* **(A)**. Masson trichrome staining; magnification x200. Arrows show interstitial fibrosis **(B)**.

The weight of the kidney was significantly higher in 5/6 Nx-3 compared to other studied groups. In rats sacrificed 1 month after nephrectomy, there were no differences in the weight of the remnant kidney (Figure 2A). We observed the abnormal shape and the atypical color of the remnant kidney in rats both 1 and 3 months after nephrectomy. Masson's trichrome staining revealed tubular injury: interstitial inflammation, fibrosis, and tubular dilation involving <25% of the field in 5/6 Nx-1 rats. In 5/6 Nx-3 group, renal tubular injury and interstitial fibrosis were significantly greater than in rats after 1 month of disease progression, showing tubular injury area between 25 and 50% of the total field (Figure 2B).

### Bone biomechanics

**Figure 3:**
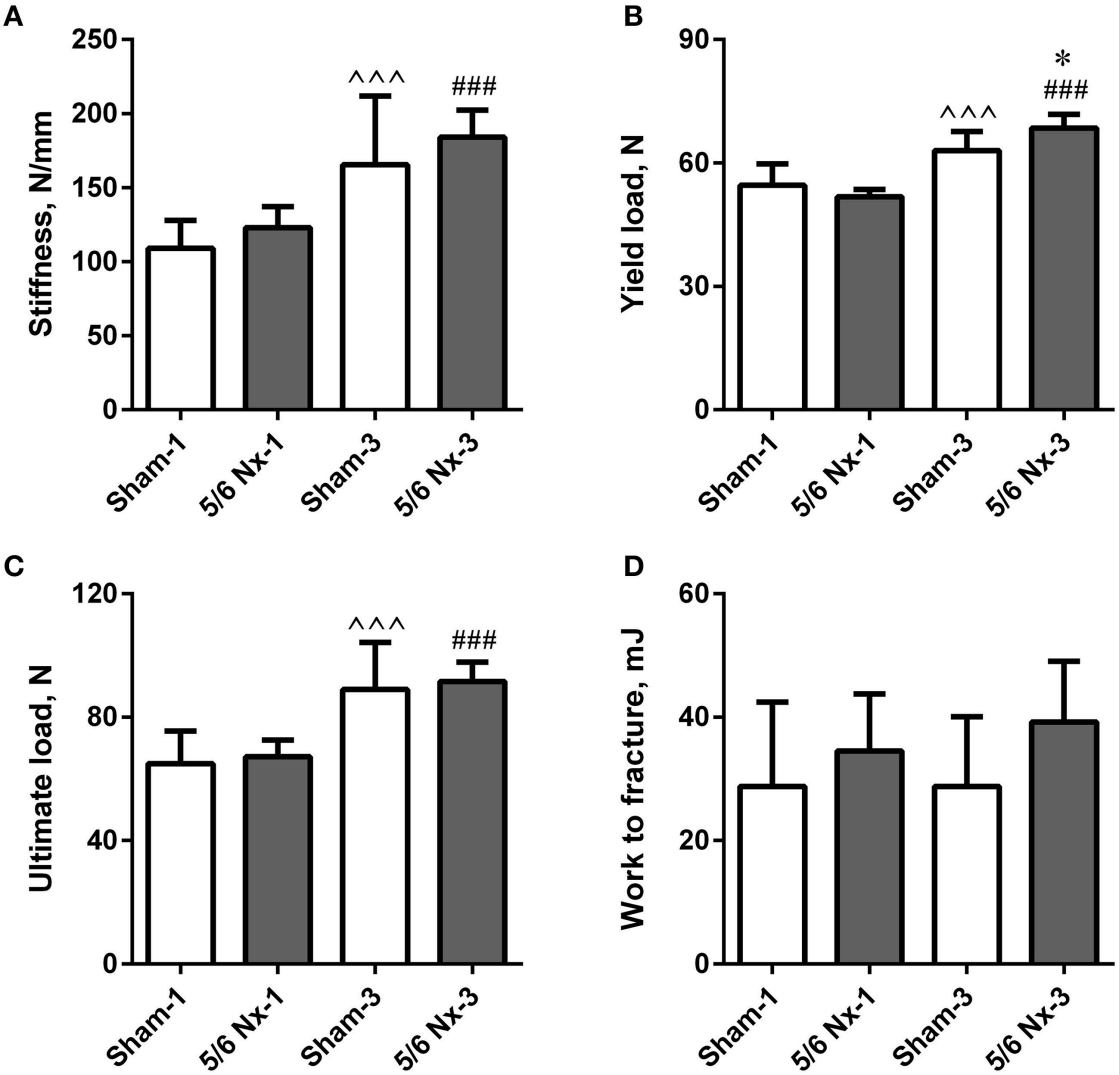
Bone biomechanics of the tibia expressed as stiffness **(A)**, yield load **(B)**, ultimate load **(C)**, and work to fracture **(D)** in sham-operated (Sham) and nephrectomized (5/6 Nx) rats after one (Sham-1 and 5/6 Nx-1) and 3 months (Sham-3 and 5/6 Nx-3) of disease progression. ^*^p < 0.05 vs. appropriate sham group; ^∧∧∧^*p* < 0.001 vs. Sham-1; ^###^*p* < 0.001 vs. 5/6 Nx-1, ANOVA with *post-hoc* Bonferroni correction. Data are mean ± *SD*.

Stiffness, yield load, and ultimate load were significantly increased in older compared to younger animals. Yield load was also significantly increased in the 5/6 Nx-3 group compared to the Sham-3 group. There were no differences in work to fracture between groups (Figure 3).

### Bone geometry

**Figure 4:**
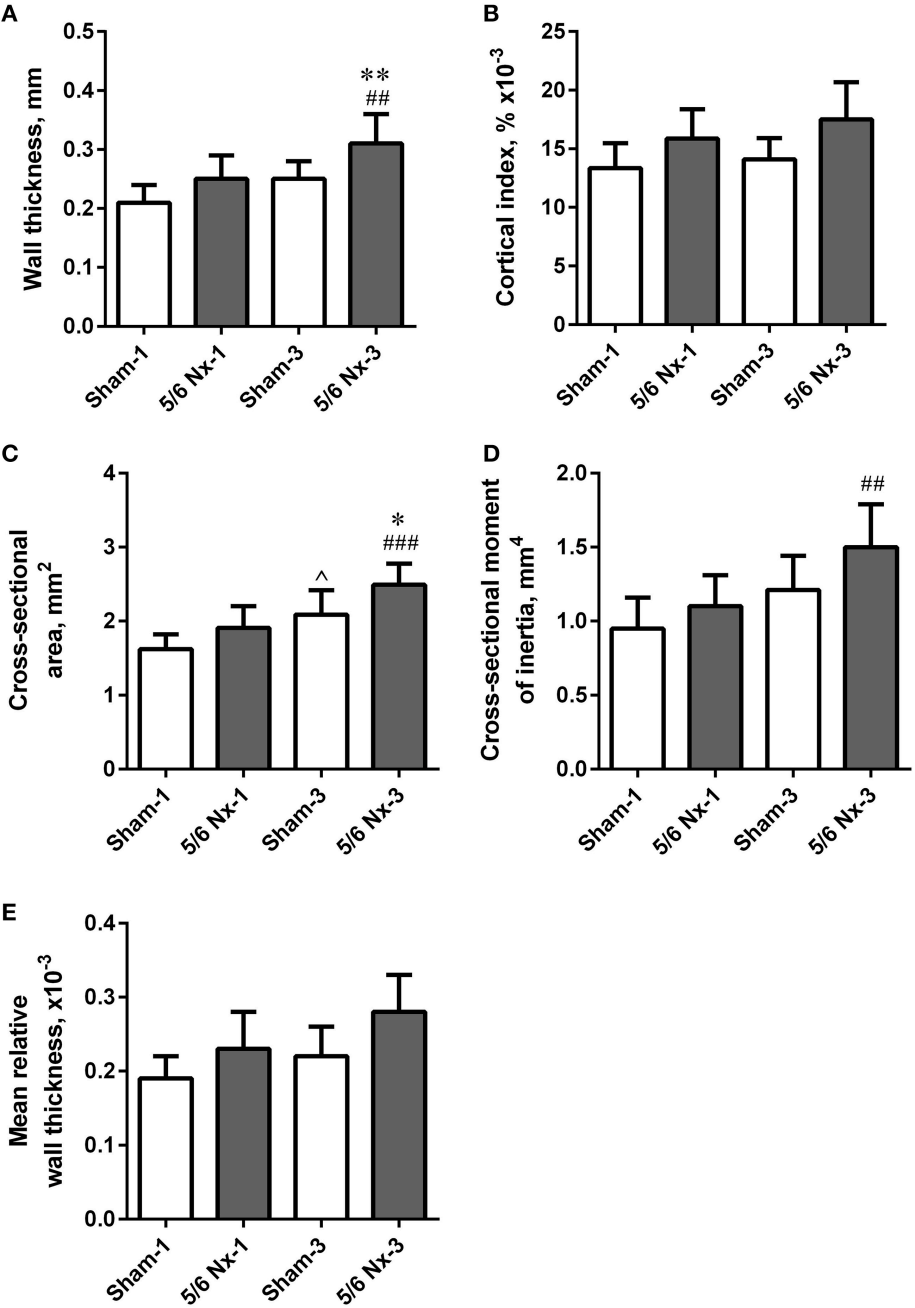
Bone geometry of the tibia expressed as wall thickness **(A)**, cortical index **(B)**, cross-sectional area **(C)**, cross-sectional moment of inertia **(D)**, and mean relative wall thickness **(E)** in sham-operated (Sham) and nephrectomized (5/6 Nx) rats after one (Sham-1 and 5/6 Nx-1) and 3 months (Sham-3 and 5/6 Nx-3) of disease progression. ^*^p < 0.05, ^**^p < 0.01 vs. appropriate sham group; ^∧^*p* < 0.05 vs. Sham-1; ^##^*p* < 0.01, ^###^*p* < 0.001 vs. 5/6 Nx-1, ANOVA with *post-hoc* Bonferroni correction. Data are mean ± *SD*.

Tibias from 5/6 Nx rats did not differ regarding length, weight, periosteal and endosteal diameters compared to the age-matched controls. Wall thickness and cross-sectional area were significantly higher after 3 months of CKD compared to age-matched controls. Wall thickness, cross-sectional area, and cross-sectional moment of inertia were significantly increased in the 5/6 Nx-3 group compared to the 5/6 Nx-1 group. Cross-sectional area was also significantly increased in the Sham-3 group compared to the Sham-1 group. There were no differences in the cortical index and mean relative wall thickness between groups (Figure 4).

### Bone mineral density based on Archimedes' method

Archimedes' principle density values of tibias were significantly higher in older compared to younger animals (1.30 ± 0.09, 1.35 ± 0.12, 1.55 ± 0.19, and 1.66 ± 0.20 g/cm^3^ in Sham-1, 5/6 Nx-1, Sham-3, and 5/6 Nx-3, respectively; *p* < 0.05, Sham-1 vs. Sham-3; *p* < 0.001, 5/6 Nx-1 vs. 5/6 Nx-3).

### Bone histology

**Figure 5:**
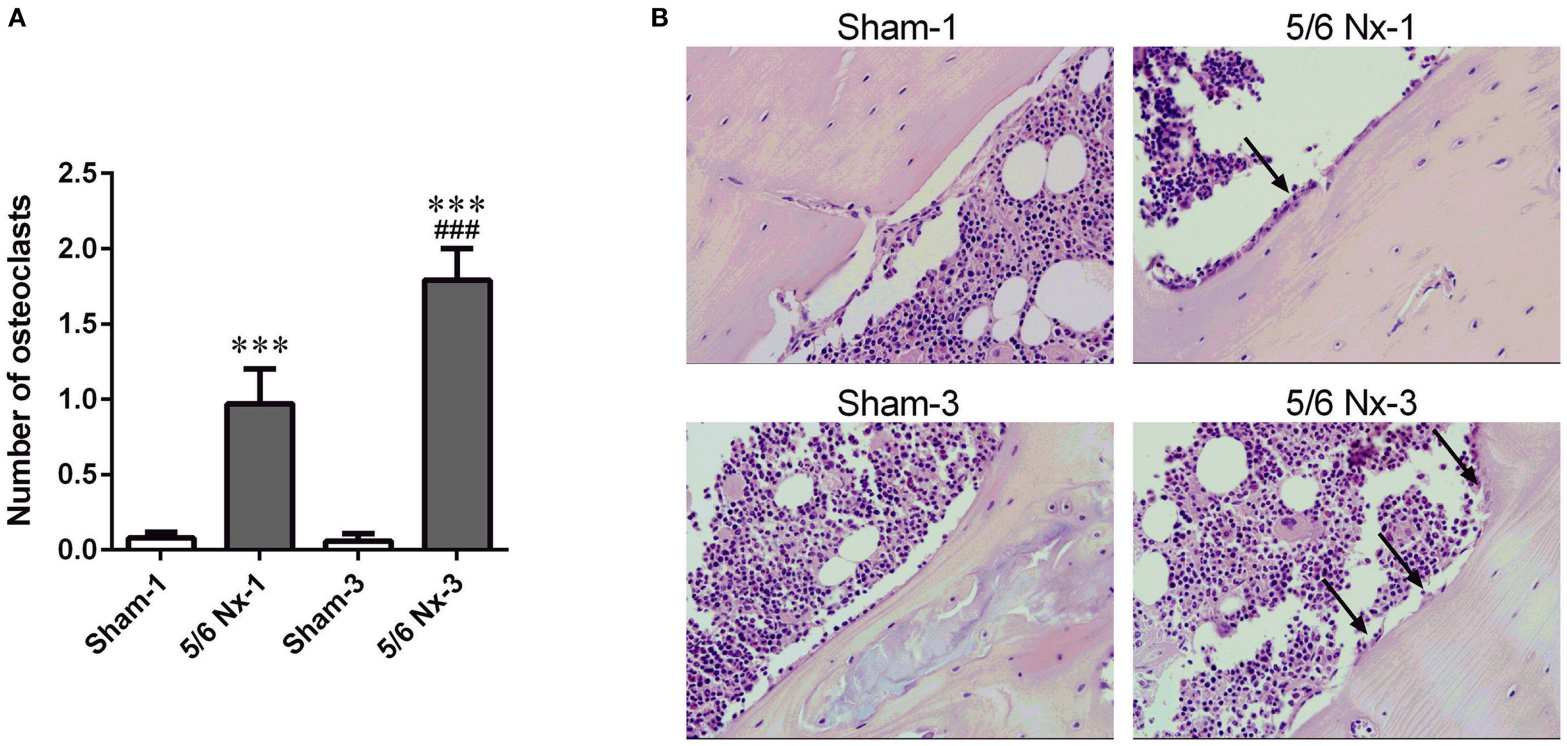
The number of osteoclasts **(A)** and representative images of the tibial cortical bone **(B)** taken from sham-operated (Sham) and subtotal nephrectomized (5/6 Nx) rats after one (Sham-1 and 5/6 Nx-1) and 3 months (Sham-3 and 5/6 Nx-3) of disease progression. ^***^p < 0.001 vs. appropriate sham group; ^###^*p* < 0.001 vs. 5/6 Nx-1, ANOVA with *post-hoc* Bonferroni correction. Data are mean ± *SD* **(A)**. Arrows indicate the increase in osteoclasts surface. Hematoxylin and Eosin staining; magnification x400 **(B)**.

Nephrectomy in rats significantly increased the number of osteoclasts per millimeter of the bone perimeter (Figure 5).

### Micro-CT measurements

Micro-CT examination of tibial cortical bone revealed a mild effect of nephrectomy on bone microarchitecture. Cortical bone density was significantly lower in the 5/6 Nx-1 group than in the Sham-1 group. Closed porosity was significantly higher in the 5/6 Nx-1 group, but significantly lower in 5/6 Nx-3 group compared to appropriate control. Additionally, cortical thickness, total cross-sectional area, cortical bone area, and cortical bone density were significantly increased whereas closed porosity was significantly decreased in older compared to younger animals after nephrectomy. Cortical thickness, total cross-sectional area, and cortical bone area were increased with age in sham-operated groups (Table 2, Figure 6).

**Table 2:** Bone architectural parameters in sham-operated (Sham) and nephrectomized (5/6 Nx) rats after one (Sham-1 and 5/6 Nx-1) and 3 months (Sham-3 and 5/6 Nx-3) of disease progression.

	**Sham-1**	**5/6 Nx-1**	**Sham-3**	**5/6 Nx-3**
BV/TV, %	46.37 ± 7.44	54.11 ± 14.90	54.38 ± 5.56	53.22 ± 4.81
Ct.th, mm	0.26 ± 0.05	0.24 ± 0.02	0.43 ± 0.05^∧∧^	0.42 ± 0.02^##^
Tt.Ar, mm^2^	7.35 ± 0.63	6.52 ± 1.68	10.58 ± 1.02^∧^	12.33 ± 1.06^#^
Ct.Ar, mm^2^	3.62 ± 0.86	3.95 ± 0.21	5.92 ± 0.15^∧^	6.31 ± 0.59^#^
Ct.Ar/Tt.Ar, %	47.82 ± 7.72	55.23 ± 15.71	55.22 ± 6.65	54.67 ± 3.90
Ma.Ar, mm^2^	3.91 ± 0.28	4.77 ± 1.59	4.86 ± 1.17	5.33 ± 1.13
BMD Ct, g/cm^3^	1.85 ± 0.04	1.66 ± 0.08^*^	1.83 ± 0.01	1.78 ± 0.02^#^
Po.V(cl), mm^3^	0.007 ± 0.003	0.010 ± 0.003	0.012 ± 0.004	0.011 ± 0.001
Po(cl), %	0.18 ± 0.04	0.25 ± 0.04^*^	0.20 ± 0.03	0.15 ± 0.02^*^^,^^##^

* p < 0.05 vs. appropriate sham group;

∧ *p < 0.05*,

∧∧ p < 0.01 vs. Sham-1;

# *p < 0.05*,

## *p < 0.01 vs. 5/6 Nx-1, ANOVA with post-hoc Bonferroni correction. Data are mean ± SD. BV/TV, cortical bone volume fraction; Ct.Th, cortical thickness; Tt.Ar, total cross-sectional area; Ct.Ar, cortical bone area; Ct.Ar/Tt.Ar, cortical area fraction; Ma.Ar, marrow area; BMD Ct, cortical bone density; Po.V(cl), volume of closed pores; Po(cl), closed porosity*.

**Figure 6:**
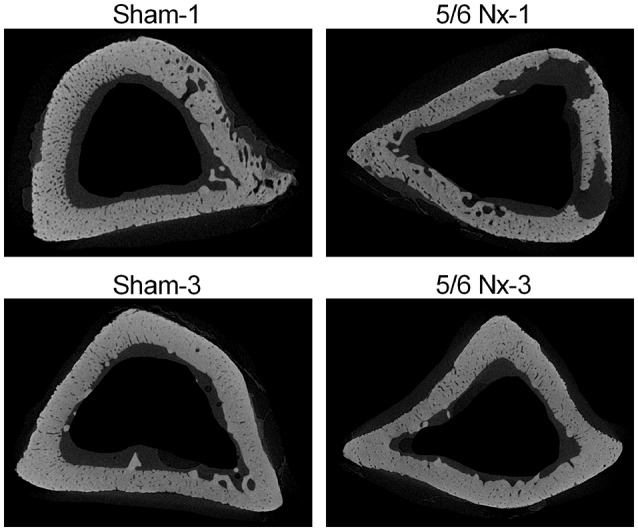
Representative micro-CT images of the proximal tibia from sham-operated (Sham) and subtotal nephrectomized (5/6 Nx) rats after one (Sham-1 and 5/6 Nx-1) and 3 months (Sham-3 and 5/6 Nx-3) of disease progression.

### Determination of serum TRP, KYN, and 3-HKYN

**Figure 7:**
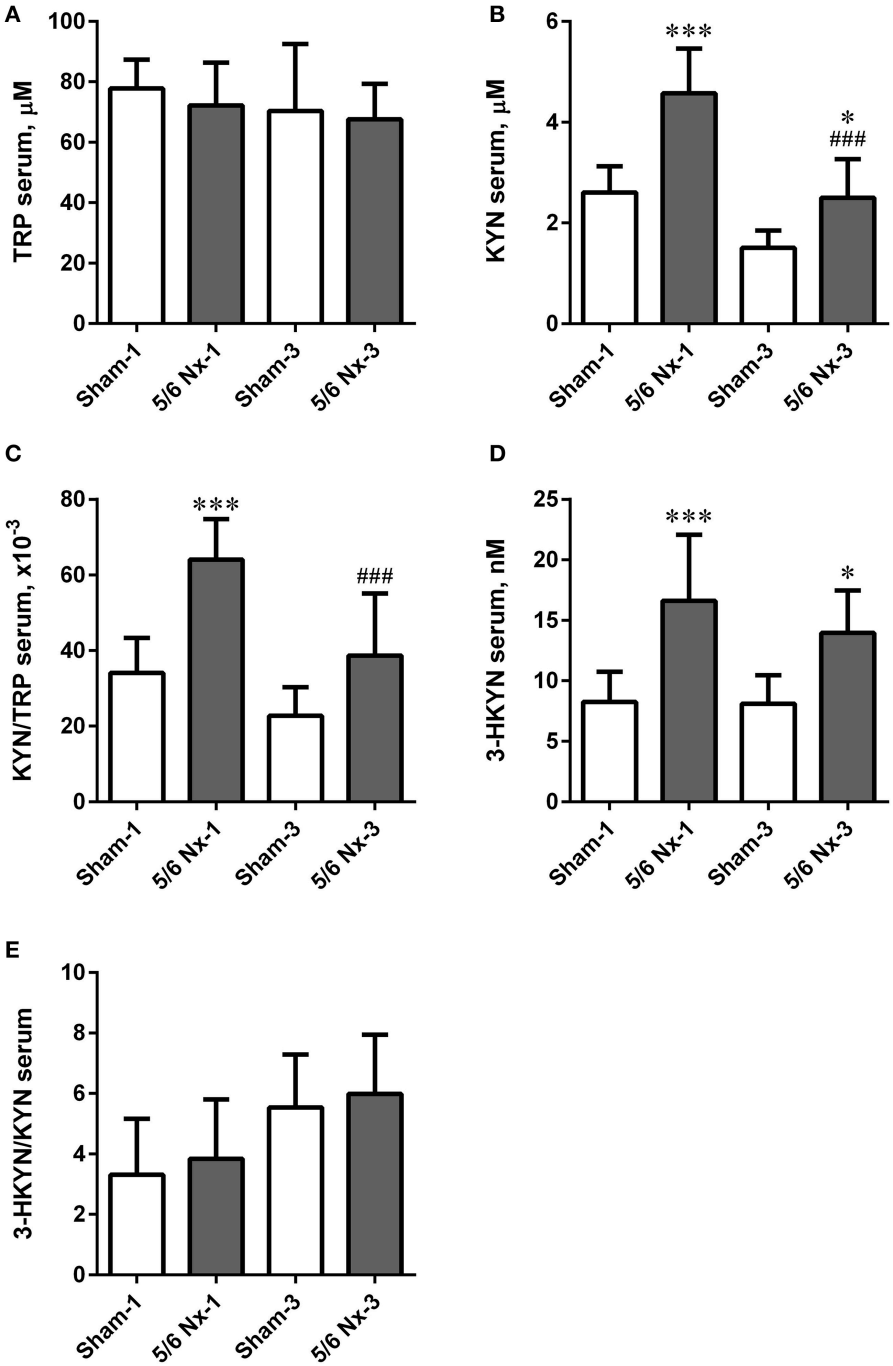
The changes in serum tryptophan (TRP; **A**), kynurenine (KYN; **B**), KYN/TRP ratio **(C)**, 3-hydroxykynurenine (3-HKYN; **D**), and 3-HKYN/KYN ratio **(E)** in sham-operated (Sham) and subtotal nephrectomized (5/6 Nx) rats after one (Sham-1 and 5/6 Nx-1) and 3 months (Sham-3 and 5/6 Nx-3) of disease progression. ^*^p < 0.05, ^***^p < 0.001 vs. appropriate sham group; ^###^*p* < 0.001 vs. 5/6 Nx-1, ANOVA with *post-hoc* Bonferroni correction. Data are mean ± *SD*.

There were no differences in serum TRP levels and 3-HKYN/KYN ratio between all studied groups. The 5/6 Nx rats were characterized by significantly higher serum KYN and 3-HKYN concentrations compared to sham-operated rats. The serum ratio of KYN/TRP was significantly higher in the 5/6 Nx-1 group compared to appropriate control. In rats sacrificed 3 months after surgery, there were no differences in serum KYN/TRP ratio. Both serum KYN concentration and serum KYN/TRP ratio were lower in 5/6 Nx-3 rats compared to 5/6 Nx-1 rats. Serum KYN concentrations tended to decrease whereas 3-HKYN/KYN ratio tended to increase with age in sham-operate groups (Figure 7).

### Determination of AHR and CYP1A1 gene expressions

**Figure 8:**
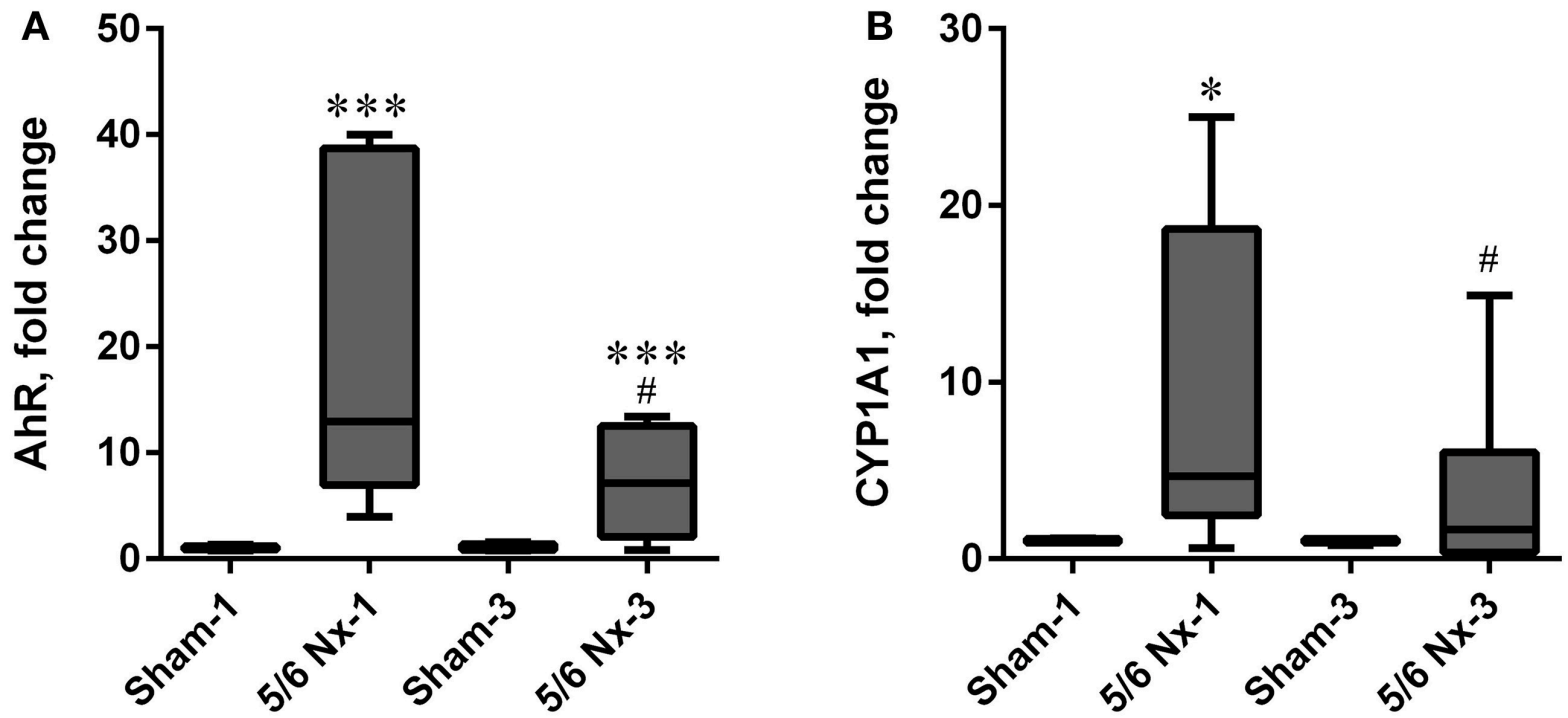
The changes in hydrocarbon receptor (AhR; **A**), and cytochrome P450 1A1 (CYP1A1; **B**) in sham-operated (Sham) and subtotal nephrectomized (5/6 Nx) rats after one (Sham-1 and 5/6 Nx-1) and 3 months (Sham-3 and 5/6 Nx-3) of disease progression. ^*^p < 0.05, ^***^p < 0.001 vs. appropriate sham group; #*p* < 0.05 vs. 5/6 Nx-1, Mann-Whitney test. Results are median (line) with interquartile range (box) and maximum and minimum values (whiskers).

The expression level of AhR was significantly increased in the 5/6 Nx groups compared to appropriate controls. The expression level of CYP1A1 was increased in the 5/6 Nx-1 group compared to appropriate control. Similarly to the serum KYN concentration and serum KYN/TRP ratio, the expression levels of AhR and CYP1A1 were lower in 5/6 Nx-3 rats compared to 5/6 Nx-1 rats (Figure 8).

### Relationships

**Figure 9:**
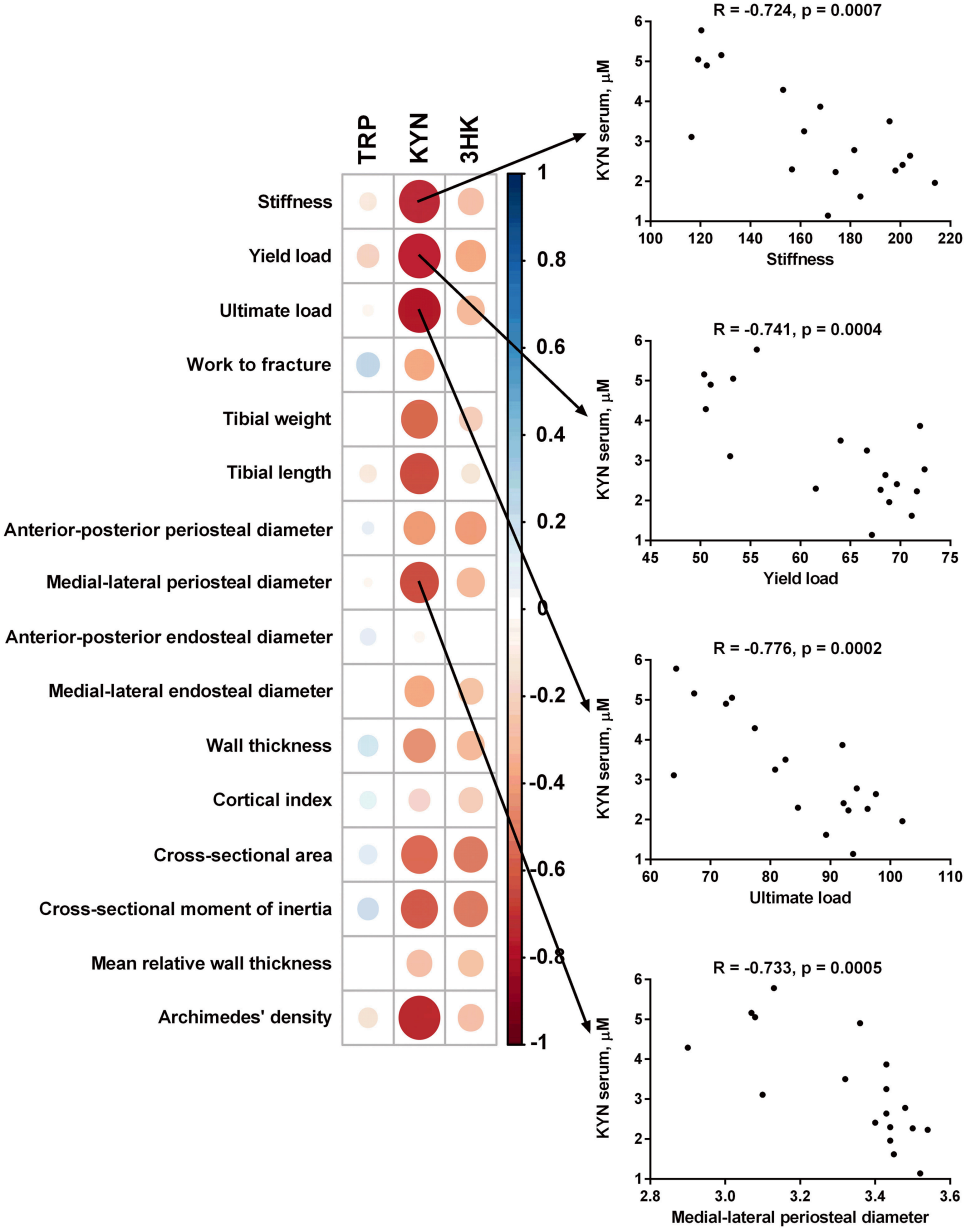
Spearman correlation matrix between tryptophan (TRP), kynurenine (KYN), and 3-hydroxykynurenine (3-HKYN) in the serum and bone properties in subtotal nephrectomized rats. The size and intensity of color represent the strength of the correlation (larger and darker circles demonstrate the strong correlation). Red colors, negative correlations; blue colors, positive correlations.

Serum level of KYN was inversely associated with stiffness, yield load, ultimate load, tibial weight, tibial length, anterior-posterior periosteal diameter, medial-lateral periosteal diameter, wall thickness, cross-sectional area, cross-sectional moment of inertia, and bone mass density of the tibia measured by Archimedes' principle method. Serum levels of 3-HKYN correlated inversely with cross-sectional area and cross-sectional moment of inertia (Figure 9).

## Discussion

We found that impaired renal function in rats with nephrectomy-induced CKD affects the peripheral kynurenine pathway, especially in the early stages of the disease and/or in the young organism. These alterations may be associated with the pathological changes in bone structure. In contrast to the central kynurenines (Kalaska et al., 2017), we observed the negative relationships between serum level of kynurenine and bone biomechanical as well as geometrical parameters. These associations may suggest unfavorable effects of peripheral kynurenine pathway metabolites on bone structure and may develop new strategies for the treatment, prevention, and diagnosis of osteoporosis in CKD patients.

The animal model induced by subtotal nephrectomy (5/6 Nx) mimics the progressive renal disease in humans and is used to assess the pathophysiological aspects and the bone structural and biomechanical changes in moderate CKD (Moscovici et al., 1996; Heveran et al., 2016). We used growing rats to better understand the bone pathophysiology at two different time points of disease progression. After 1 month of disease, the rats aged 2 and a half months were growing rapidly. In children with CKD, the growing skeleton is extremely dynamic and at particular risk of deterioration (Schmitt and Mehls, 2011). After 3 months of disease, the rats were at the beginning of a period of bone maintenance with the onset of skeletal maturity. These age-related influences are critical to serum differences in the bone turnover biomarker. Similarly to others (Zappitelli et al., 2013), we observed age-dependent decrease of serum ALP, a bone formation marker. In addition to measuring the activity of ALP, the KDIGO guidelines recommend controlling of serum phosphorus, calcium, and PTH beginning in patients with moderate CKD (Moe et al., 2009). Phosphorus and calcium levels are relatively stable even in severe CKD. However, nearly 60% of patients with moderate CKD have elevated PTH levels (Levin et al., 2007). We observed the similar serum phosphorus and calcium concentrations in 5/6 Nx animals and appropriate controls. The 5/6 Nx rats presented higher serum PTH levels. In patients with CKD, maintaining of sodium/potassium balance is crucial for the prevention of CKD-related complications (He et al., 2016). In our study, serum potassium concentrations tended to increase in nephrectomized animals 3 months after surgery. Additionally, the 5/6 Nx rats showed higher concentrations of creatinine and urea nitrogen compared to healthy controls. The level of creatinine was significantly higher in older compared to younger animals with CKD, which may indicate progression of the disease. Changes in above parameters may mimic mild or moderate CKD (Jokihaara et al., 2006; Moe et al., 2009; Heveran et al., 2016).

Moderate CKD impairs bone microarchitecture and decreases bone material quality in male C57Bl/6J mice after 5/6 nephrectomy (Heveran et al., 2016). Patients with moderate CKD have a two-fold increase in fracture prevalence compared with age-matched controls (Naylor et al., 2014). Patients over 70 years of age with moderate CKD have a two-fold increase in fracture-related mortality (Nitsch et al., 2009). The bone strength in the course of CKD can be quantitatively measured by the biomechanical analysis based on the bone strength, stiffness and ability to absorb energy (Turner and Burr, 1993; Turner, 2006). The three-point bending test is commonly used to measure the bone properties of the cortical bone in rodents and other small animals (Oksztulska-Kolanek et al., 2016). In our study, stiffness, yield load, and ultimate load were significantly increased in older compared to younger animals. However, comparing nephrectomized and sham-operated rats, there were no significant changes in the main biomechanical parameters. Yield load was even significantly increased in the 5/6 Nx-3 group compared to the Sham-3 group. Several studies assessed the bone quality in animals with subtotal nephrectomy using biomechanical tests (Jokihaara et al., 2006; Iwamoto et al., 2012; Heveran et al., 2016); the findings of these studies were in agreement with our results. The unexpected biomechanical data suggest that the early adaptive response related to growth in the very young, rapidly growing rats could provide protection from the deleterious effects of the moderate CKD on the cortical bone strength. Long bones have spatial dimensions which define their geometrical properties. Although our geometrical analysis was calculated based on not very precise measurements with calipers, we obtained consistent and statistically significant results. Interestingly, the wall thickness and cross-sectional area were also significantly higher after 3 months of CKD compared to age-matched controls. The increase in wall thickness and the cross-sectional area provides the evidence for extensive bone modeling in nephrectomized animals and can point to geometrical adaptations (Jokihaara et al., 2006) that is especially evident in growing skeleton (Turner et al., 1987). In our study, the changes of bone geometry could affect the bone strength in nephrectomized animals. The cortical part of the bone is mainly responsible for mechanical properties. Cortical bone strength and geometry have been evaluated by using not only mechanical testing but also a histological examination and micro-CT measurements (Particelli et al., 2012). Histological examination is the “gold standard” for the diagnosis of renal osteodystrophy (Moe et al., 2009). In the present study, nephrectomy significantly increased the number of osteoclasts in rats both 1 and 3 months after surgery. Although the histological results confirmed that nephrectomy was associated with an excessive number of osteoclasts in bone (Teitelbaum, 2000), the marker of bone resorption—serum TRACP 5b, remained statistically unchanged. Osteoclast number is generally considered a reliable index of bone resorption, and circulating levels of TRACP 5b are often used as a marker of systemic bone resorption (Halleen et al., 2000). However, neither higher osteoclasts abundance nor the increased activity of TRACP 5b does not fully reflect an increased rate of bone resorption (Seeman and Nguyen, 2016). Increased number of osteoclasts can be the result of direct calcium deficiency or PTH stimulation (Thompson et al., 1975; Jilka et al., 2010). The results similar to ours were previously described by Kuroshima et al. (2012), who noticed that serum TRACP 5b did not reflect the suppressed bone resorption status in mice with long-term zoledronic acid therapy. Moreover, in a mouse model of estrogen-deficient osteoporosis, the higher osteoclast activity was recorded in risedronate-treated groups compared with that in the vehicle. The authors explain this phenomenon by the mechanism of the more vigorous bone remodeling process, which occurred in risedronate-treated than in untreated animals (Nam et al., 2012). Cortical bone strength is not only dependent on the cross-sectional geometry of the bone but is also related to microstructural parameters (Currey, 1988; Yeni et al., 1997; Wachter et al., 2002). Closed porosity was significantly higher in rats 1 month after nephrectomy, but significantly lower in rats 3 months after nephrectomy compared to appropriate control. Cortical bone density was also significantly increased in older compared to younger animals after nephrectomy. These results suggest that bone microarchitecture in rapidly growing rats is very susceptible to metabolic changes caused by renal impairment. In rats 3 months after nephrectomy, renal tubular injury and interstitial fibrosis were significantly greater than in rats after 1 month of disease progression, showing tubular injury area between 25 and 50% of the total field. Interestingly, the weight of the remnant kidney was significantly elevated in 5/6 Nx-3 compared to both 5/6 Nx-1 and Sham-3, and serum blood urea nitrogen concentration was not increased but even showed tendency to decline during this time, suggesting that kidney hypertrophy allowed for partial compensation of kidney function and correction of unfavorable changes in bone microarchitecture and strength.

The mechanisms responsible for bone disturbances in the early stage of CKD remains unknown. Serotonin, a monoamine derived from tryptophan, may play a potential role in bone metabolism (Yadav et al., 2009; Ducy and Karsenty, 2010; Ducy, 2011; Wang et al., 2014). It has been shown that reducing the peripheral serotonin levels enhanced the bone strength and bone mineral density in the ovariectomized animals (Wei et al., 2014). Recently, we demonstrated the association between peripheral serotonin metabolism and bone biomechanical properties in growing 5/6 Nx rats. The impaired kidney function in these animals affected the peripheral serotonin metabolism, which was associated with the pathological changes in bone structure (Pawlak et al., 2016). What is worth noting, only 1% of tryptophan is used for serotonin synthesis, but as much as 95% is metabolized *via* the kynurenine pathway (Stone, 1993).

In the present study, we observed the altered peripheral kynurenine metabolism in rats with CKD. Serum concentrations of KYN and its direct, highly reactive metabolite−3-HKYN rose significantly in the 5/6 Nx group. The elevation in peripheral kynurenines levels in the course of CKD is consistent with the previous clinical and experimental data (Saito et al., 2000; Pawlak et al., 2001a,b). We also observed the significantly higher serum KYN/TRP ratio in 5/6 Nx-1 animals compared to controls. KYN/TRP ratio reflects the activity of indoleamine 2,3-dioxygenase, the enzyme that stimulates the conversion of TRP to KYN. Increased indoleamine 2,3-dioxygenase activity and elevated serum levels of kynurenines indicate intensified peripheral kynurenine turnover and may primarily be a consequence of chronic inflammation in the course of CKD (Schefold et al., 2009). Serum KYN concentrations tended to decrease whereas 3-HKYN/KYN ratio tended to increase with age in sham-operate groups. These changes could have resulted in unchanging 3-HKYN concentrations in sham-operated groups.

We found the substantial role of the augmented peripheral kynurenine turnover in bone strength in growing rats with nephrectomy-induced CKD. Serum level of KYN was inversely associated with stiffness, yield load, ultimate load and main geometrical parameters in 5/6 Nx rats. These findings suggest that elevated peripheral KYN may reduce bone strength and stiffness. The potential explanation for these relationships includes the interaction of kynurenine with AhR in tibias of studied animals. AhR is a cytosolic receptor for several low molecular weight exogenous and endogenous molecules that can control bone homeostasis in a receptor activator of NF-κB ligand (RANKL)/c-Fos-dependent manner (Izawa et al., 2016). The most widely recognized exogenous AhR agonist is tetrachlorodibenzodioxin (TCDD) (Mandal, 2005). Interestingly, kynurenine has been identified as one of the endogenous AhR agonist (Opitz et al., 2011). Exposure of wild-type mice to TCDD resulted in mechanically weaker bones, harder bone matrix, and thinner and more porous cortical bone. Only a few minor effects were seen on bone mechanical properties and morphology in AhR knockout animals (Herlin et al., 2013). Moreover, AhR knockout mice exhibited an increased bone mass and decreased bone resorption (Yu et al., 2015). These results suggest that the altered bone properties are highly dependent on the functional AhR. In our study, we determined the gene expression of AhR and AhR-dependent CYP1A1. The gene expression of both AhR and CYP1A1 was significantly increased in the 5/6 Nx-1 group compared to appropriate controls and was lower in 5/6 Nx-3 rats compared to 5/6 Nx-1 rats. Our results suggest that the elevated peripheral kynurenine level in the course of CKD may cause the pathological changes in bone structure *via* AhR pathway. We also do not exclude that there are other pathological mechanisms of kynurenine action on bone metabolism (Michalowska et al., 2015). In CKD, elevated levels of peripheral kynurenine can affect the RANKL/osteoprotegerin axis and histone deacetylase-3 or runt-related transcription factor 2 expressions (El Refaey et al., 2015; Refaey et al., 2017). We also do not exclude that this phenomenon was induced by AhR ligands except for kynurenine. There are many ligands of AhR in the serum of CKD patients, for example indoxyl sulfate (Schroeder et al., 2010). However, the gene expression of AhR in the bone tissue correlated positively with serum kynurenine concentration and was inversely correlated with the main parameters of bone biomechanics, bone geometry, and bone mass density. Above potential mechanism appears to be particularly pronounced in the early stage of nephrectomy induced-CKD. Three months after 5/6 nephrectomy, both serum KYN concentration and AhR expression are decreasing compared to the early stage of CKD. These changes are accompanied by the hypertrophy of the remnant kidney and consequently bone “remodeling.”

**Figure 10:**
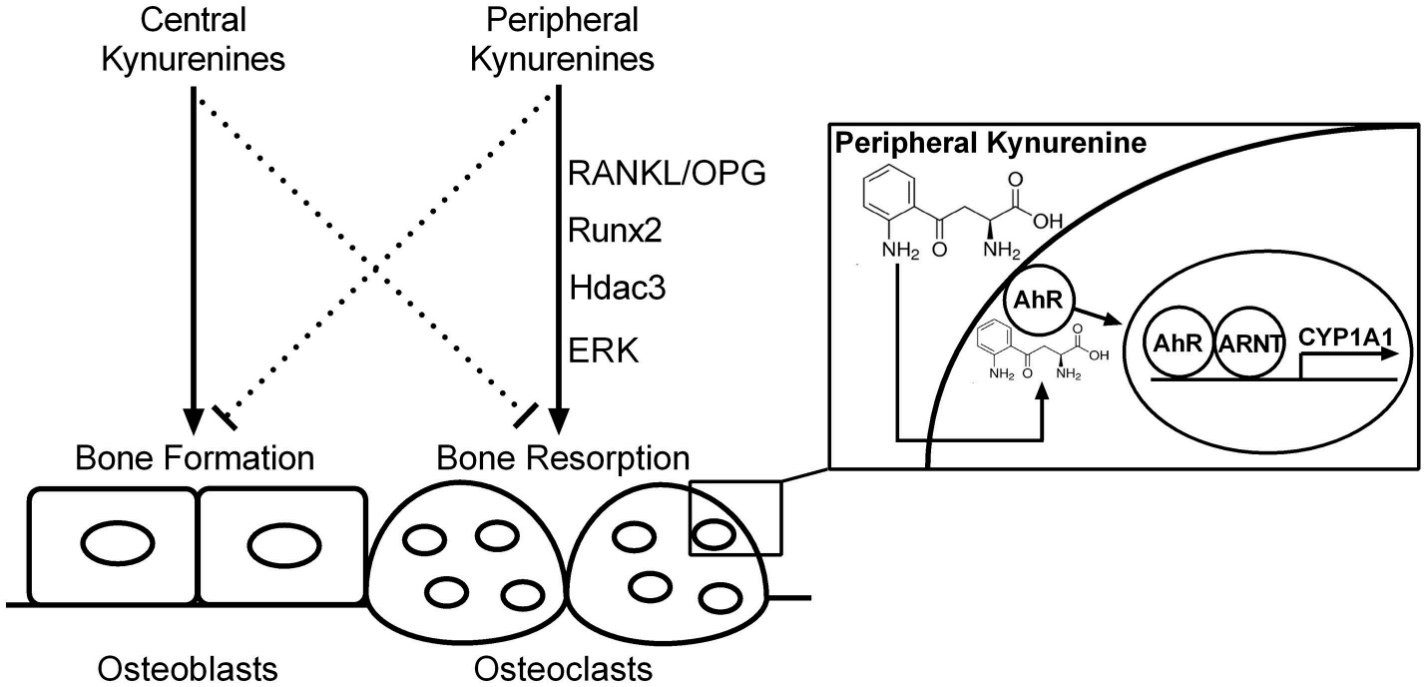
The hypothetical scheme of central and peripheral kynurenine-dependent regulation on bone remodeling based on our data and the results of other authors (El Refaey et al., 2015; Refaey et al., 2017). RANKL/OPG, receptor activator of NF-κB ligand/osteoprotegerin axis; Runx2, runt-related transcription factor 2; Hdac3, histone deacetylase-3; ERK, extracellular signal-regulated kinases; AhR, aryl hydrocarbon receptor; ARNT, aryl hydrocarbon receptor nuclear translocator protein; CYP1A1, AhR-dependent cytochrome P450 1A1; solid line, activation; dotted line, inhibition.

Peripheral kynurenine pathway is not autonomous but is linked to the brain kynurenines (Schwarcz et al., 2012). The effect of kynurenine on bone metabolism, similarly to serotonin (Ducy and Karsenty, 2010), seems to be dependent on the site of its synthesis. Peripheral kynurenines may decrease bone formation/increase bone resorption while brain-derived kynurenines may exert opposite influences on bone formation (Figure 10). Further studies could examine the similarities, differences, and relations between the peripheral and central action of kynurenine on bone metabolism.

In conclusion, our study for the first time demonstrates the association between intensified peripheral kynurenine turnover, AhR activation, and impaired bone microarchitecture and strength in growing rats with mild to moderate CKD. The observed associations may give grounds to the development of new strategies for the diagnosis, treatment, and prevention of osteoporosis in CKD patients.

## Author contributions

Conceived and designed the experiments: BK, KP, and DP. Performed the experiments: BK, KP, TD, EO, BZ, AR, JR, PL, MD, AP, and DP. Analyzed the data: BK, KP, MB, and DP. Contributed reagents/materials/analysis tools: BK, KP, TD, and DP. All authors took part in drafting the work, revising it critically and approved all parts of the work.

### Conflict of interest statement

The authors declare that the research was conducted in the absence of any commercial or financial relationships that could be construed as a potential conflict of interest.
